# Community engagement and involvement in developing a community-based signposting tool for mental distress

**DOI:** 10.1186/s40900-026-00939-9

**Published:** 2026-07-09

**Authors:** Claire Wicks, Cara Booker, Meena Kumari, Susan McPherson

**Affiliations:** 1 School of Health and Social Care, University of Essex, Colchester, CO4 3SQ UK; 2 Institute of Social and Economic Research, University of Essex, Colchester, CO4 3SQ UK; 3 Institute of Public Health and Wellbeing, University of Essex, Colchester, CO4 3SQ UK

**Keywords:** Public involvement, Involvement practices, Tool development, Mental distress, Community-based

## Abstract

**Background:**

Common mental health conditions are increasing in England, with growing severity and unmet need. Evidence shows that mental wellbeing is strongly shaped by wider social determinants such as income, housing, employment, and relationships. Conventional clinical approaches may not adequately address these underlying factors, highlighting the need for preventative, community-based solutions.

**Aim:**

This paper describes the community involvement and engagement (CEI) approach used to develop CoSign, a digital signposting tool designed to help individuals identify social determinants contributing to mental distress and access appropriate community support.

**Involvement and engagement approach:**

The development of CoSign incorporated CEI throughout the research process, guided by the INVOLVE research-cycle framework and the IAP2 Spectrum of Public Participation. A total of 29 stakeholders—including healthcare professionals, voluntary sector representatives, policymakers, and individuals with lived experience—contributed through workshops, consultations, and collaborative activities. Engagement focused on shaping the tool’s design, content, accessibility, and implementation considerations.

**Activities and impact:**

Stakeholder input informed key aspects of CoSign, including the identification of relevant domains of need, refinement of language to improve accessibility, and the structure of the user experience. Practical considerations, such as service capacity, inclusivity, and potential barriers to engagement, were highlighted and addressed. The resulting web-based tool enables users to self-identify areas of social need and receive tailored signposting to community resources. CEI enhanced the relevance, acceptability, and usability of the tool.

**Conclusion:**

Meaningful community involvement is essential in developing accessible, preventative mental health tools grounded in real-world needs. However, challenges such as limited resources, stakeholder fatigue, and equity in participation must be managed. The CoSign project demonstrates the value of CEI while underscoring the importance of flexible, context-sensitive approaches to engagement.

## Abstract (plain language summary)

This paper describes the development of a community-focused tool called CoSign, designed to help people understand how social and economic factors may be affecting their mental wellbeing and guide them to appropriate support. Mental health problems are increasing in England, and many people struggle to access care. The paper highlights that mental health is strongly influenced by “wider determinants” such as income, housing, relationships, employment, and social conditions. In some cases, medical treatments alone may not address these underlying causes. Community-based approaches, such as social prescribing, aim to fill this gap but still often require contact with health services and may not fully meet people’s needs. CoSign was created to help individuals identify the social factors contributing to their distress and find community support early, before problems worsen. It is an online questionnaire that asks users about areas like work, housing, and social life, then suggests relevant services. The tool focuses on prevention rather than diagnosis. A key feature of the project was strong community involvement. Researchers worked with 29 stakeholders, including health professionals, community organisations, and people with lived experience. Their input shaped the tool’s design, ensuring it was practical, accessible, and relevant. For example, stakeholders emphasised clear language, inclusivity, and avoiding overwhelming users. The paper concludes that involving communities throughout the research process improves the usefulness and accessibility of mental health tools. However, it also highlights challenges such as limited resources, stakeholder fatigue, and balancing meaningful engagement with practical constraints.

## Introduction

Prevalence of common mental health conditions (CMHCs) in England’s general population continues to increase [1]. This trend is evident across most age groups and in men and women. Since 2014, the severity of CMHCs has also increased indicating that not only are more people living with poor mental health, but the level of suffering is also greater [2]. For people wishing to seek treatment, mental health services are continuing to struggle to meet the increasing demand [3], while many experience barriers to accessing health care [4]. Both scenarios result in a significant number of people who are living with an untreated and/or unrecognised mental health need [5].

Social determinants of mental health can be defined as “…income, employment, socioeconomic status, education, food security, housing, social support, discrimination, childhood adversity, as well as the neighbourhood social and physical conditions in which people live” [6]. These factors are also sometimes referred to as “wider determinants”, particularly in the context of UK local authority public health initiatives seeking to address factors that may contribute to worsening public mental health. In their extensive review, Kirkbride and colleagues [6] detail the extent of high quality evidence demonstrating the link between these social, structural factors and population mental health and how the most marginalised populations often face intersectional, multiple disadvantage in respect of these determinants. The review sets out the evidence supporting various hypotheses such as the bidirectional relationship between socioeconomic disadvantage and poor mental health; and the potential biological, sociological and psychological pathways [6]. The review also sets out the scale of the impact in various domains, for example that children growing up while exposed to socioeconomic disadvantage are 2–3 times more likely to experience mental health difficulties than their peers. In cases where wider determinants are exacerbating or causing mental distress, the effectiveness of medicalised approaches including talking therapies and pharmacology may be limited to providing coping strategies or masking the underlying problem. Indeed, the ethics of prescribing pharmaceutical interventions for social issues has been widely critiqued [7].

The UK National Health Service (NHS) 10-Year health plan for England [8], recognises the opportunities offered by moving towards a model of community-based healthcare, including reducing demand on overstretched mental health services. Social prescribing emerged from community-based initiatives as a model to refer people experiencing social problems or mild mental health conditions to community-based support based on the individual’s interests and preferences. However, referral (including self-referral) to social prescribing still requires contact with a health provider. Furthermore, people who have been prescribed medication may experience impaired emotion regulation making social interactions more difficult which impedes participation in social interventions [9]. It could be experienced as empowering for individuals to identify their own needs and find support; as well as an early intervention approach, preventing escalation of difficulties which would otherwise lead to the need to access NHS care and/or living with an unmet mental health need, for example, on a waiting list.

Several tools which take into account the wider determinants of mental health have previously been developed in the UK to measure outcomes or to assess need in primary and secondary care settings, e.g. Dialogue+, Outcomes Star, and the Live Well Social prescribing tool. However, consultation with local health professionals and a search of published literature did not identify any existing tool that has been designed specifically to support an individual to identify the wider determinants that may be triggering their current distress and to facilitate signposting to appropriate community-based support. Here we briefly consider two of the commonly used assessment and outcome tools in England’s health services, Dialogue + and Live Well.

Dialogue+ [10] is being used in several NHS Trusts in England to guide conversations and monitor outcomes in adult community mental health services. It assesses life satisfaction through asking about satisfaction with eight aspects of life, with an additional six questions about treatment satisfaction. As a measure, Dialogue + was developed to capture subjective quality of life and treatment experience, rather than clinical symptoms or mental distress. Whilst life satisfaction can be associated with mental health [11], low satisfaction cannot be assumed to indicate mental distress. In line with this distinction, Priebe and Golden [12] report weak correlation between Dialogue’s eight quality of life items and subscales of the Positive and Negative Syndrome Scale (*r*=-20 to − 0.37) which measures positive, negative and general mental distress symptoms.

Live Well is a social prescribing tool used in England which assesses practical, social, and emotional needs of people accessing social prescribing services [13]. The tool supports the implementation of recommendations outlined in the National Institute for Health and Care Excellence (NICE) guideline on mental wellbeing and independence in older people and was endorsed by NICE in July 2017 [14]. Proven useful for social prescribers, the Live Well tool identifies areas of life where a person may need some support. Response options are “yes” or “no” throughout, with some weighting applied to the scoring of certain items. Responses are used to develop an action plan based on the “Five ways to Wellbeing” [15]. However, the Live Well tool is not underpinned by empirical research or psychometric approaches and there is no evidence to indicate the questions asked or responses given are comprehensively addressing wider determinants nor associated with mental distress.

In collaboration with community mental health service providers and commissioners as well as people with lived experience of mental distress, the authors of the current paper undertook an applied research project which used secondary analysis of population data to develop “CoSign”, a tool which enables individuals to consider the wider determinants which may be impacting their mental wellbeing and support them to identify support which might target these wider determinants. A separate paper [16] describes the technical development process including the methodology, data, ethical approval and statistical analysis approach.

CoSign is a web-based questionnaire-style tool for use by individuals experiencing emotional distress and can be used on any device connected to the internet. It comprises three components; (1) Four screening questions which determine which of the main question blocks will be shown (employment status, relationship status, caring responsibilities and whether the individual has any immediate family); (2) The main question block (maximum 31 questions), asking users about wider-determinants of mental health e.g. housing, their job, their family, their partner, their social life etc.; (3) Signposts to local services and/or national advice and information. The signposts direct users to services which are countywide or available nationally where countywide services do not exist or where national advice is most relevant. Users are also directed to additional search tools maintained by local organisations which can use their postcode to find additional localized support. Whilst CoSign asks users questions about their personal situation, it is not intended to assess or diagnose mental health conditions. Rather its purpose is to identify areas of an individual’s social, economic or environmental context which may be particularly challenging and hence triggering current emotional distress. This information and the subsequent signposts generated by CoSign can be used by individuals to access community-based services that are able to offer practical support to tackle the issue. The intention is to support people in community settings before emotional distress escalates towards mental health conditions requiring more formal treatment services and is therefore designed to offer a tool in the domain of preventative mental health.

CoSign was developed in Essex, a coastal county in the East of England and currently the signposting component of the tool is relevant only to adults living in Essex. However, the screening questions and the core set of 31 items are applicable to all UK residents and the tool could be readily adapted for other counties by introducing a new set of signposts specific to the new geographical area. Essex faces significant mental health challenges, shaped by socioeconomic inequalities, rurality, and areas of coastal deprivation [17]. A 2023 analysis found that of 5,000 community voluntary sector services identified in Essex, 51% reported increased demand particularly in relation to mental health support, and 74% reported increasing complexity of demand such as mental health challenges intersecting with economic hardship [18]. As such, a signposting tool focusing on wider determinants to facilitate improved prevention in the community sector is highly relevant to public health initiatives in the county.

The current paper describes the approach to Community Involvement and Engagement (CEI) in the research. Over the past two decades, there has been increasing recognition of the importance of engaging communities in health research to enhance relevance, trust and impact. This trend has been driven in the UK by policy initiatives promoting public and patient involvement (e.g. INVOLVE established in 1996, NIHR, 2015) and internationally by the growth of community-based participatory research [19, 20]. CEI is one approach used to engage communities in research. The key characteristics of CEI includes involving a broad range of individuals or groups who have a vested interested in the problem being addressed and recognises multiple levels of influence and expertise across stakeholders [21]. In addition, the involvement of stakeholders can vary across the lifecycle of the research and range from informational (e.g. advice) to empowerment (e.g. shared decision making) [23]. CEI aims to enhance the practical relevance, acceptability and update of research findings by incorporating diverse perspectives early on. CEI was an integral component of CoSign’s development and is therefore described here in detail, separately from the technical paper which describes the research undertaken to develop the content of CoSign [16]. The aim of this paper is to describe the CEI activities undertaken in the development of CoSign.

## Involvement and engagement approach

### Engagement techniques

The need for a signposting tool based on wider determinants of mental health itself derived from prior engagement work with the researchers’ local community mental health sector. Once the need for the overall piece of work was established, stakeholder engagement in the project was planned using the INVOLVE research‑cycle involvement framework [24], which outlines opportunities for public involvement across the stages of research. The INVOLVE framework was developed for primary care and health research, and explicitly connects involvement levels to research stages and acknowledges that involvement can vary by stage.

Descriptions of the INVOLVE Research‑Cycle Involvement Stages are described below with the practical application outlined in Table 1. “Deciding What to Do” ensures that the proposed research reflects shared priorities and is grounded in community-based needs. “Deciding How to Do It” provides space for public contributors to influence the research design and methods. “Doing It” involves stakeholders directly carrying out aspects of the research (this stage is separated into distinct phases in Table 1). “Letting People Know the Results” ensures public contributors help communicate findings. The signposting tool was developed as a prototype ready to pilot in this project but not evaluated. As such, this latter stage will be revisited in a future pilot and evaluation. “Knowing What to Do Next” translates in this project to public contributors helping interpret findings and plan future steps, including a pilot and evaluation.

In addition, the IAP2 Spectrum of Public Participation [23], was used to identify appropriate levels of participation for each group across the stages of research. All engagement activities were guided by the UK Standards for Public Involvement to ensure that engagement was inclusive, supportive and collaborative [25].

**Table 1:** Stakeholder involvement matrix for the community signposting tool

	Project Activity	Lived Experience Advisors	Practitioners (NHS/VCSE)	Local Government / policy advisors
Deciding what to do	**Identifying need:** Iterative involvement over 30 months and across multiple contacts with approximately 87 stakeholders highlighted recurring themes that demonstrated a clear need for further targeted support. This included prior project work guided by stakeholders which focussed on reducing local mental health and care inequalities, a community‑based workshop, seminar attendance and knowledge exchange activities.	*n* = 7	*n*=approx.80 across various activities.	
Deciding how to do it	**Develop protocol**: Research team identified a practical workstream and appropriate analytical processes within the boundaries of project resources. **Presentation**: All local stakeholders were invited to attend a presentation outlining the project. The project proposal was presented for feedback on the viability of the tool in practice and the proposed methodological approach.	INFORM *n* = 4	INFORM *n* = 11	INFORM *n* = 4
Doing it: Identifying key words	**Interactive task**: Organisational stakeholders at the initial meeting, and Lived Experience Advisors took part in an exercise to identify key words—drilling down into the finer details—related to the wider determinants of mental health.	COLLABORATE *n* = 4	COLLABORATE *n* = 11	COLLABORATE *n* = 4
Doing it: Categorising	**Refining key words**: The project team refined the list of key words, with the removal of: duplicates, key words related to individual factors, issues that could not be modified with community-based support, applied specific adult populations (e.g. older adults), could not be mapped against at least one question from the Understanding Society questionnaires. **Grouping key words**: The remaining 55 key words were sorted into categories by stakeholders (those who attended the initial meeting were invited to participate) via an online task. The key words could be grouped into a maximum of 8 categories. Stakeholders were encouraged to name the categories.	COLLABORATE *n* = 4	COLLABORATE *n* = 11 This task was completed anonymously.	
Doing it: Developing themes	**Identifying similarities between categories**: The project team merged 69 categories into broad themes based on commonality of key words. This resulted in 10 themes. **Refining themes**: Lived experience advisors were asked to review the themes, by assessing the key words included. Two themes were merged resulting in 8 final themes. Other stakeholders were informed.	COLLABORATE *n* = 4	INFORM *n* = 5	INFORM *n* = 1
Doing it: Analytical processes	**Selection of key words and UKHLS items**: Factor analysis was used to confirm the association between the potential UKHLS items and the key word, and the association between the key word and overarching theme. Multiple regression was used to test the association between each key word and mental distress using the GHQ Likert and caseness scales. Machine learning was used to verify the findings. All keywords were included into one mode with mental health to identify the key words that ‘remain’. These processes are reported in full elsewhere. Key words that were not eliminated during these processes were considered for inclusion with the best fitting UKHLS item (analytical and face validity) selected for each key word.	INFORM *n* = 4	INFORM *n* = 5	INFORM *n* = 1
Doing it: translating research findings into the tool	Lived Experience Advisors asked to provide feedback on the language used in the signposting tool. Including introductory information, example questions and signposting responses.	COLLABORATE *n* = 4	INFORM *n* = 6	INFORM *n* = 6
Letting people know the results	**Presentation and discussion**: All local stakeholders were invited to attend a final meeting. A recap of the developmental process was shared along with details of the signposting tool: flow of survey, an example question and response options for each theme, criteria for signposting and example signposting messages.	INFORM *n* = 4	INFORM *n* = 6	INFORM *n* = 6
Knowing what to do next	**Discussion**: Stakeholders provided input into a range of possible next steps, including further refinements needed, evaluation of the tool - experience of using the tool and its effectiveness, e.g. which services do people go on to access and how does their mental distress change), potential for linking the signposting tool to other systems and monitoring use (e.g. accessing other systems following signposting), format of the tool.	CONSULT *n* = 4	CONSULT *n* = 6	CONSULT *n* = 6

The research adopted the following definitions proposed by Concannon and Meissner [21]. “Stakeholder” was defined as an individual or group who is responsible for or affected by health- and healthcare-related decisions that can be informed by research evidence. “Engagement” was defined as a bi-directional relationship between the stakeholder and researcher that results in informed decision-making about the selection, conduct, and use of research.

The level of CEI varied across stages of the research as shown in Table 1. The levels of engagement included “inform” (to provide the public with objective and balanced information to assist them in understanding the problem, alternatives, opportunities and/or solutions); “consult” (to obtain public feedback on analysis, alternatives and/or decisions); “involve” (to work directly with the public throughout the process to ensure that public concerns and aspirations are consistently understood and considered); and “collaborate” (to partner with the public in each aspect of the decision including the development of alternatives and the identification of the preferred solution). Whilst the levels may be intended for use at project level, it was not feasible to work at the level of “collaborate” across all stages of the project. While this was the ideal, we have reported the levels obtained for transparency. Decisions around involvement were determined by both the resources available to the project and pragmatic considerations, including stakeholder fatigue and the project’s timeline dictated by the availability of funding.

A lived experience advisor was invited to offer feedback on their experience being involved in the project. A selection of their comments are included in the discussion. In addition, they were invited to review and provide comments on the manuscript prior to publication.

### Stakeholder recruitment

Recruitment of community organisational representatives was via a snowballing approach. An email was sent to an established mailing list of known community mental health providers and partners who were asked to share the information with anyone else who might be interested in participating. The mailing list included partners from NHS, Voluntary Community Social Enterprise and local authority sectors and had approximately 80 members. The email included an outline of the project aim and commitment and a brief animated video about why the research was needed. In addition, an update on the research was provided in a bi-annual stakeholder newsletter produced by the research team. This welcomed engagement with the project at any stage to increase awareness, support for or potential uptake of the tool, and impact.

A separate information sheet was produced in plain English language to invite lived experience advisors to participate in the research. The University of Essex Service User Research Group and Healthwatch Essex mental health ambassador group were asked to share the information sheet. In addition, the information was posted on social media channels such as LinkedIn. Five expressions of interest were received with four lived experience advisors joining the project. The remaining person was subsequently unable to participate for personal reasons.

### Ethical considerations

The CEI approach was guided from the outset by the Health Research Authority’s decision tool [26] and the University of Essex Ethical Public Engagement tool [22]. The Health Research Authority’s decision tool asks three questions about the project to determine whether it is considered research, as defined by the UK Policy Framework for Health and Social Care Research. The questions ask whether participants are randomised into different groups, whether the study protocol demands changing treatment/care/services from accepted standards for any of the patients/service users involved, and whether the study was designed to produce generalisable or transferable findings. In relation to CoSign, all answers were “no” indicating that the project would not be considered research by the NHS. The University of Essex Ethical Public Engagement tool sets out activities such as public outreach, communications, patient involvement, collaborative research and community engagement as distinct from research. Using the self-assessment decision making tool, it was established that the CEI activities described in this paper did not constitute research needing research ethics review for a variety of reasons including that the stakeholders were acting as consultants or advisors rather than participants; they took part in meetings generating ideas; they did not need to meet specific eligibility criteria; no data were collected from them and no personal or other data about stakeholders were recorded; stakeholders were invited based on their expertise and their views were not formally analysed as data; they were involved in decision making during the activities; their insights were used to inform and guide the research process; their views and opinions were used as feedback and not as data to generate new knowledge. Principles of ethical engagement were followed throughout the process including transparency about the purpose of the stakeholder meetings; not collecting or storing any personal data; ensuring appropriate technology was used to enable stakeholders to participate equally; explaining the time commitment and timeline from the outset. Attendance at stakeholder meetings was entirely voluntary. The role of stakeholders was purely consultative and did not constitute research study participation. Meetings with stakeholders were not recorded, transcribed or analysed; slides used to present information at the meetings were shared with members of the wider network whether they attended the meeting or not to enable multiple forms of engagement with the project.

In relation to the technical research undertaken to develop the tool, the project itself used data from waves 9–14 of *Understanding Society*: the United Kingdom Longitudinal Study [27]. This study has ethical approval from the University of Essex Ethics Committee by dated letter for waves 9–11, and ethics approval reference ETH1920-0123 for Wave 12, ETH2021-0015 for Wave 13, and ETH2122-0246 for Wave 14. Use of the data is described elsewhere in a technical paper [16].

## Activities and impact

### Engagement and activities

A total of 29 stakeholders (numbers varied across activities), including mental health practitioners, community workers, local policymakers and people with lived experience took part in CEI activities across the research project. With the exception of two activities where only lived experience advisors contributed, all stakeholders were invited to contribute equally.

### Early themes and insights

Stakeholders emphasised important practical considerations not only about how the tool might work, but about the services to which people might be signposted. Stakeholders highlighted the importance of ensuring services existed to provide support in areas of need identified by the signposting tool. In addition, they highlighted the possibility that existing services may receive an increase in demand resulting from local use of the tool and this needed to be taken into consideration. Stakeholders helped forge connections between relevant partners in the community, e.g. where there existed relevant community asset maps and referral platforms to help build the signposting component of the tool. Lived experience advisors raised issues including the language to be used, e.g. avoiding jargon; ensuring accessibility of the tool (e.g. considering digital exclusion, translation, people with hearing/visual impairments); ensuring that services signposted were appropriate, e.g. service entry criteria; and avoiding re-creating barriers that the tool was attempting to overcome, e.g. feeling excluded from care/services where sexual orientation options are not fully inclusive in demographic questions.

### Impact on the research

An initial phase of the research involved generating a long list of potential keywords reflecting wider determinants of mental health which could be mapped to question items in the dataset being used for population level statistical analysis. This long list was generated by stakeholders in a wide-ranging discussion using Padlet (an online visual collaboration tool used for group based creative ideas generation) to capture views on how to interpret and operationalise the concepts underpinning the term “wider determinants”. The initial long list of items later subject to statistical analysis was therefore directly generated through stakeholder input. Another key aspect of the research process involved clustering individual items reflecting wider determinants of mental health into themes which would then be used to structure further stages of the statistical analysis [16]. This task, which is similar to a qualitative technique of forming categories from codes, was undertaken primarily by stakeholders, and fine-tuned by the research team with reference to relevant literature. This ensured that the clusters that emerged had face validity, with construct validity confirmed via analytical processes (see [16]). Following these initial stages of research, informed by the CEI activities, statistical analysis using the population dataset went on to further narrow down the list of items to those which were highly associated with mental health [16]. Jointly the CEI activities informed the central elements of research and statistical analysis which generated the final list of 31 core items in the tool.

Following this final stage of statistical analysis, CEI activities moved on to reviewing the final list of items, checking the wording, testing the prototype tool which incorporated these items and providing insights to help fine tune the layout and user experience aspects of the tool. One issue discussed in depth by stakeholders at this stage was whether, for those users with multiple social issues, being presented with a very long list of signposts might be overwhelming. Options were considered such as limiting the overall number of signposts shown or asking users to select areas to focus on. Unanimity was not reached on this issue with some stakeholders feeling the benefits of having the complete list covering all areas outweighed other considerations and other stakeholders taking the opposite view. The final version of the tool therefore represents a majority rather than a unanimous view. It was agreed that the pilot would explore the issue in more depth including qualitative interviews which would ask users specifically about whether any aspects of using the tool felt overwhelming.

In terms of initial stakeholder insights noted above relating to potential increase in demand for services, this was also taken account of in the pilot and evaluation design. As a result, the evaluation will ask users which services they accessed in order to understand the impact of CoSign on local services. Evaluation findings will be fed back to members of the stakeholder group which includes local commissioners and service managers.

## Discussion

This paper describes how CEI was employed in the development of a signposting tool to assess potential mental distress due to social, emotional and environmental need, and direct to appropriate community-based support.

### Engagement methods and practical barriers

Engaging with stakeholders from the beginning of the project gave confidence that the tool was grounded in real community needs and supported the project aim of creating an accessible resource for all adults. In addition, it ensured that the research and development process was shaped by the perspectives of those who would ultimately use or support the tool, rather than being driven solely by assumptions of the research team. This early involvement helped surface practical considerations, potential barriers, and priorities that might otherwise have been overlooked, therefore strengthening the tool’s relevance across the wider community and reinforcing the project’s commitment to equitable, community‑centred support. One of the lived experience advisors for the project provided further reflections into how the development process was experienced and the project commitment to meaningful involvement. They noted “throughout the project I felt my comments and opinions were very much listened to. I could clearly see where, directly, opinions and suggestions were taken on board and used. I would extend this further and say I saw other examples, from colleagues where this happened. This project was definitely one where it not only felt co-produced but you could see it happening in real time.”

To increase accessibility for geographically dispersed stakeholders across a large English county with relatively poor transport infrastructure between urban, rural and coastal areas, CEI meetings were held remotely via Zoom. This likely increased the number of stakeholders able to participate by reducing travel requirements and other practical barriers to attendance. However, consistent with findings from community-engaged research conducted during and after the COVID-19 pandemic, online engagement may simultaneously create new forms of exclusion for those with limited access to technology, internet connectivity or digital literacy [28]. CEI activities such as online tasks and posing specific questions for discussion were employed to enhance the richness of involvement. The brief and highly focused tasks using Padlet with anonymous posting achieved the highest levels of engagement throughout the project, potentially reflecting evidence that online environments can lower participation barriers for some individuals. Nevertheless, the virtual format limited opportunities for informal interaction and relationship-building that often occur before, during and after face-to-face meetings.

Previous research suggests that trust, connection and group cohesion require more deliberate facilitation in online settings, with practitioners often needing to create dedicated opportunities for social interaction and collective care. While the decision to hold online meetings was intended to improve accessibility and broaden participation, it may also have reduced participants’ sense of connection to this particular project. However, as noted previously, this particular CEI work was an extension of existing engagement work with an established broader network of partnerships and collaborations between University mental health researchers and the local mental health sector. This broader engagement context includes a wide variety of online and face-to-face interactions between individuals, teams, groups and other research projects including mental health research seminars at the University and both generic and project specific stakeholder events held in local venues. Ultimately, online meetings may have favoured breadth of participation over depth of engagement in this specific project but because existing relationships and connections already existed this would have been mitigated to an extent. Future phases of tool development will explore opportunities for face-to-face engagement with stakeholder groups, recognising that hybrid approaches may offer a means of combining the accessibility benefits of online participation with the relationship-building and trust development associated with in-person engagement.

The requirement from research funding bodies to include CEI as an essential component of research is growing. This has radically increased the demand on local organisations by universities and other research institutions to participate in research. Key local organisations and their representatives are at risk of experiencing “participation fatigue” from ongoing demands to participate in research activities. This may diminish both engagement and buy-in to individual projects [29]. We attempted to manage this by minimising the time commitment to just three meetings across the development stage while also offering alternative ways to contribute through receiving slides and updates and inviting individuals to send feedback and insights directly to the research team. As the project was research intensive, this approach was deemed appropriate but may also have resulted in it being overshadowed by other projects with more regular or recent meetings. We attempted to find a balance between too frequent meetings which may have exhausted already fatigued stakeholders, with fewer meetings without too much time passing between communications or meetings to ensure that stakeholders would still feel invested and involved.

### Structure, equity and commitment

A formalised arrangement or commitment was not established with organisational representatives and they were not paid for their time since they attended meetings as part of their professional roles. Whilst a formal arrangement may have increased level of buy-in, e.g. approval from management that an agreed amount of time would be spent on the project and/or feeling recognised as an equal member of the research team, a formal commitment may have dissuaded participation. The light touch approach employed to engagement meant that attendance at meetings fluctuated, which limited the engagement process to an extent. The stakeholders who attended infrequently likely did not feel fully invested or informed about the research as it developed. This may have resulted in some stakeholders feeling less able to fully contribute when they were in attendance, although at each meeting there was a full recap on the development work to date and all stakeholders were part of a wider mailing list who received information and slides used in stakeholder meetings, whether or not they attended.

In contrast, the lived experience advisors were recruited via a slightly more formal process and were paid for their time. They therefore participated more consistently and may have felt more invested in the project and observed where their contributions were influencing development. A lived experience advisor reflected on what helped their involvement meaningful, “Regular meetings, good clear communication, knowing my opinion could make a meaningful difference and that lived experience was not just a tick box, but a fundamental aspect of the project.”

Although different approaches to payment of professionals versus lived experience advisors aligns with standard practices, it raises questions about transparency, equity and how differing reward structures may influence perceived value and participation dynamics in co-production, especially when working with charity sector partners who have limited resources to cover staff time for non-core activity. It is likely the organisational representatives may not have been aware that the lived experience advisors were being financially compensated for their time, since this information was not explicitly provided. In addition, stakeholders may bring both organisational and lived experience insights. How stakeholders are positioned within the project (as professionals or experts by experience; paid or unpaid) may influence how they engage or feel comfortable engaging and whether power hierarchies are reflected in the process. Both issues may lead to concerns around fairness or power imbalances.

A useful distinction emerged when reflecting on the difference between broad community involvement and the more formal model often seen in funding applications, where one or two community partners act as co-applicants and receive partial funding. The co-applicant model can offer clear advantages, including deeper partnership with specific organisations, shared ownership of project outcomes, and the ability to resource community partners for their time and expertise. However, it can also narrow the range of voices involved, privileging the perspectives of funded partners over the wider community. In contrast, inviting a broad range of stakeholders without payment enabled a more diverse set of experiences and viewpoints to shape the tool, while also raising challenges around equity, accessibility, and the risk of excluding those unable to contribute unpaid time. Ultimately, funding available can limit the options researchers have to provide a range of forms of reward, thanks and payment and in this context the nature of the funding meant that there was very limited resource for CEI activities compared to resources allocated to CEI on more formal research grants.

### Trust, collaboration and decision‑making

Stakeholder engagement can be a delicate balancing act, especially at initial stages when the goals and potential impact may not yet resonate with all parties. Building trust and demonstrating relevance early on is critical to fostering meaningful engagement. While asking stakeholders for formal commitments can initially feel off-putting; particularly if the project relevance is not yet fully established, it can also serve to elevate the perceived importance of both the work and their role in it. Formalising involvement, whether through agreements or defined responsibilities, signals that stakeholder contribution is valued and that the project is structured and purposeful. However, this approach requires careful timing and sensitivity; trust and clarity around mutual benefits should ideally be established first to avoid deterring engagement. Done correctly, this level of formality can move stakeholder participation from passive consultation toward genuine collaboration. In the current project, no such formal agreements were in place. Further, as part of the research process, some final decisions were made by the research team independently either for pragmatic reasons or because some decisions required evidence and statistical expertise to be prioritised. These decisions included deciding on the thresholds for signposting, identifying the services being signposted to, and writing the signposting messages (with some exemplar feedback from lived experience advisors). The research team maintained trust with stakeholders through transparency about any technical decisions made.

## Conclusion

This paper has outlined the practical, structural, and relational aspects of Community Engagement and Involvement (CEI) in applied health research, developing a statistically reliable community-based signposting tool addressing mental distress linked to wider determinants. The case study demonstrates that meaningful CEI is essential for ensuring that tools intended to support public mental health are relevant, accessible, and grounded in real-world need while maintaining research rigour.

The findings highlight broader challenges associated with CEI, including balancing meaningful engagement with limited resources; managing increasing demands placed on community partners; maintaining inclusive and equitable participation; and ensuring involvement remains meaningful throughout while balancing the need for certain technical decisions to be based on evidence and statistical expertise. As expectations for CEI continue to grow across research funding and practice, there is a need for flexible, context-sensitive approaches that reflect local circumstances and constraints.

Overall, the project illustrates that CEI can enhance both the quality and applicability of research outputs when implemented thoughtfully. However, it also underscores the importance of ongoing reflection, transparency, and adaptation in engagement processes. Future work should focus on refining approaches to CEI to ensure they remain both effective and sustainable, while supporting strong partnerships between researchers and communities.

## Data Availability

Not applicable.

## Abbreviations

CEI: Community engagement and involvement
CMHCs: Common Mental Health Conditions
NHS: National Health Service
NICE: National Institute for Health and Care Excellence
UKHLS: The United Kingdom Longitudinal Study
VCSE: Voluntary Community Social Enterprise
